# Circularly permuted GTPase YqeH binds 30S ribosomal subunit: Implications for its role in ribosome assembly

**DOI:** 10.1016/j.bbrc.2009.06.078

**Published:** 2009-09-04

**Authors:** Baskaran Anand, Parag Surana, Sagar Bhogaraju, Sushmita Pahari, Balaji Prakash

**Affiliations:** Department of Biological Sciences and Bioengineering, Indian Institute of Technology, Kanpur 208016, India

**Keywords:** Ribosome assembly, cpGTPase, RNA chaperone, GTP hydrolysis, Circular permutation, Strand dissociation, Strand annealing, RNA folding, Ribosome binding, RNA binding

## Abstract

YqeH, a circularly permuted GTPase, is conserved among bacteria and eukaryotes including humans. It was shown to be essential for the assembly of small ribosomal (30S) subunit in bacteria. However, whether YqeH interacts with 30S ribosome and how it may participate in 30S assembly are not known. Here, using co-sedimentation experiments, we report that YqeH co-associates with 30S ribosome in the GTP-bound form. In order to probe whether YqeH functions as RNA chaperone in 30S assembly, we assayed for strand dissociation and annealing activity. While YqeH does not exhibit these activities, it binds a non-specific single and double-stranded RNA, which unlike the 30S binding is independent of GTP/GDP binding and does not affect intrinsic GTP hydrolysis rates. Further, S5, a ribosomal protein which participates during the initial stages of 30S assembly, was found to promote GTP hydrolysis and RNA binding activities of YqeH.

## Introduction

Circularly permuted GTPases (cpGTPases) represent a class of GTPases that display a curious circular permutation in the order of sequence motifs [1,2]. They represent various domains of life and are categorized into four subfamilies represented by proteins *Escherichia coli* YjeQ (RsgA), *Bacillus subtilis* YlqF (RbgA) and YqeH and *Saccharomyces cerevisiae* YawG [1,2]. In the past, efforts to elucidate the role of cpGTPases have shed light on their likely function in ribosome biogenesis [3–9]. YjeQ (RsgA) interacts with the 30S subunit of ribosome in presence of a GTP analog and it was shown that the GTPase activity is stimulated by 30S binding [3,5]. NOG2 and LSG1 are eukaryotic members of YawG subfamily and associate with the 60S pre-ribosomal particle [6,7]. YlqF (RbgA) participates in the late step of 50S subunit assembly in *B. subtilis* and its GTPase activity too was found to be stimulated by 50S binding [8,9].

YqeH, representing one of the four subfamilies of cpGTPases and distributed across bacterial and eukaryotic genomes including humans [1,2], was shown to participate in 30S biogenesis in *B. subtilis* [10,11]. An ortholog of YqeH in *Arabidopsis thaliana* was initially reported to be a plant Nitric Oxide Synthase (AtNOS1) [12]. It was later renamed as Nitric Oxide Associated protein (AtNOA1), since the NOS activity could not be reproduced [13–15]. Recently, it was confirmed to be a functional cpGTPase and not a Nitric Oxide Synthase [16]. In addition, studies on plastid encoded RIF1, an ortholog of YqeH in *A. thaliana*, shows that deletion of *rif1* gene impairs plastome encoded protein synthesis [17]. Interestingly, it was observed that YqeH from *B. subtilis* heterologously restores the mutant phenotype of *rif1*. These studies strengthen the view that YqeH performs a ribosome associated function. However, unlike other cpGTPase subfamilies such as YjeQ(RsgA) and YlqF(RbgA), direct association between YqeH and ribosomal subunits was not reported and the precise role of YqeH in ribosome assembly remains elusive. Here, we show that YqeH co-associates with the 30S ribosomal subunit and that this association is more stable in presence of GTP or its non-hydrolysable analog GDPNP. We also find that the N and C-terminal domains in this protein, mediate ribosome/RNA binding. Unlike YjeQ(RsgA) and YlqF(RbgA), a distinctive feature of YqeH is that the ribosome/RNA association does not influence its GTP hydrolysis rates. On the other hand, in line with 30S binding, we observe increased GTP hydrolysis rates in the presence of S5, a ribosomal protein known to participate in the early stages of 30S assembly. We also find that YqeH binds single and double-stranded RNA in a nucleotide independent fashion. We envisaged an RNA chaperone like activity for YqeH; however, we find that it does not exhibit RNA strand dissociation and annealing activities.

## Materials and methods

*Co-sedimentation assay.* GST-tagged YqeH and its deletion constructs, GST-YlqF and His-S5 were cloned, overexpressed and purified to homogeneity. Crude ribosomes were purified from *B. subtilis*. Briefly, a reaction mixture, consisting of crude ribosomes (*A*_260_ = 2), 500 nM protein (GST-YqeH or its derivatives; GST-YlqF; GST) and 1 mM nucleotide (GTP/GDP/GDPNP), and incubated at 37 °C for 30 min, was layered on a linear sucrose gradient (18–50%). Following centrifugation at 90,000*g*, samples were fractionated and resolved using SDS–PAGE. The presence of the proteins was detected using an anti-GST antibody in a Western blot. For details refer to Supplementary material.

*Nucleotide binding and GTP hydrolysis.* The nucleotide binding experiments were carried out with 5 μM YqeH (or GST) and 200 nM mant-GDP or mant-GDPNP (Jena Biosciences) as detailed in Supplementary material. After incubation, the fluorescent nucleotides were excited at 355 nm and emission (at 448 nm) was monitored between 400 and 600 nm using a spectrofluorimeter (Perkin-Elmer).

The hydrolysis of GTP into inorganic phosphate (Pi) was measured in a calorimetric Malachite green assay [18]. The reaction mixture (50 μl), consisting of 375 nM YqeH (or its derivatives) and 400 μM GTP was incubated for a given time and the absorbance at 630 nm was measured. To probe the influence of RNA and S5 on GTPase activity, the M assays additionally contained 2 μl of 1 mg/ml single or double-stranded RNA and 375 nM S5, respectively. For details refer to Supplementary material.

*Electrophoretic mobility shift assay.* EMSA was performed with 5 μM proteins (YqeH or its derivatives; GST; S5), 1 mM of nucleotides (GTP/GDP) and 1 μl of labeled (7000 cpm/μl) single or double-stranded RNA, incubated at 37 °C for 30 min before stopping the reaction. RNA was resolved in 12% native PAGE. The gel was dried and exposed to phosphor imager screen (Kodak) for visualization. For details, see Supplementary material. For annealing activity, the experiment was performed with complementary ssRNAs that were not pre-annealed. Equal amounts of both ssRNAs were added to increasing concentrations of YqeH (2.5, 5, 10 μM) and incubated for 2 h at 37 °C.

## Results

### YqeH interacts with 30S ribosomal subunit

**Fig. 1:**
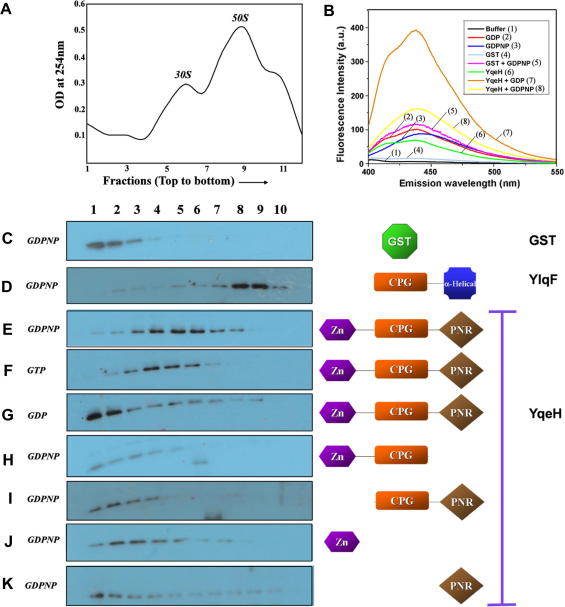
YqeH binds 30S ribosomal subunit in the presence of GDPNP/GTP. (A) Co-sedimentation experiments were carried out by incubating purified ribosomes with GST-YqeH (or GST-YlqF) and nucleotides. Following centrifugation, the absorbance at 254 nm was monitored for the fractions collected (see Supplementary material for details). A representative ribosome profile thus obtained is shown here and peaks corresponding to 30S and 50S subunits are marked. Peaks corresponding to fractions 6 and 9 contain 30S and 50S subunits (see Supplementary material: Fig. Sl). (b) Emission spectra (400–550 nm) of fluorescent mant-GDP and mant-GDPNP excited at 355 nm are shown with and without YqeH, as displayed in the inset. The fluorescence intensity is shown in arbitrary units (a.u.). (C–K) Fractions collected following co-sedimentation experiments with GST, GST-YlqF and GST-YqeH or its derivatives, were probed using anti-GST antibody in a Western blot. Shown on the right side of the gels (C–K) are the proteins used, indicating the domains they possess. (C) Purified GST was used as a negative control, as all constructs carry an N-terminal GST tag. (D) YlqF, which interacts with 50S, was used as a marker to identify the 50S fractions and as a positive control. Deletion constructs (H) ΔC-YqeH (residues 1–224) and (I) ΔN-YqeH (residues 64–366) and the stand-alone Zn finger (residues 1–46) domain (J) and PNR (residues 225–366) domain (K) are also indicated. The fractions corresponding to the ribosome profile are shown on the top and the nucleotides (GDP/GTP/GDPNP) used are indicated on the left. A high stoichiometric ratio of nucleotide (1 mM) to protein (500 nM) was used to ensure the desired nucleotide bound state.

For three of the four cpGTPase subfamilies, i.e., YjeQ (RsgA), YlqF (RbgA) and YawG, a direct interaction with the ribosomal subunits was shown [3–9]. However, similar studies have not been reported for YqeH, although it was shown to be involved in 30S assembly [10,11]. Further, domain assignments of YqeH predicted that the N-terminal region consists of treble-clef Zn-finger domain while the C-terminal domain was not characterized until lately [2]. A recent structural analysis of YqeH from *Geobacillus stearothermophilus* (gsYqeH), identifies the C-terminal domain to be a peptide/nucleotide recognition (PNR) domain [19]. This domain was found to be structurally homologous to tryptophan RNA-binding attenuation protein (TRAP) and the residues important for RNA binding in TRAP were found to be conserved in the PNR domain of YqeH as well [19]. Further, the N-terminal treble-clef Zn-finger domain is also present in ribosomal proteins S14 and L24E [2], further strengthening the view that YqeH may bind the ribosome. Therefore, using co-sedimentation experiments (see Materials and methods) we tested if YqeH would interact with ribosome. Further, to probe the effect of GTP/GDP binding on ribosome binding, experiments were conducted in the presence of GDP, GTP and GDPNP (non-hydrolysable GTP analog). To ascertain that the nucleotides do bind the protein, fluorescent nucleotide binding to YqeH was examined using mant-GDP, mant-GDPNP. An increased fluorescence of the mant nucleotides in presence of YqeH suggests their binding to the protein (Fig. 1B). Interestingly, a higher affinity for GDP than for GTP was suggested for gsYqeH and AtNOA1 [16,19]. Hence, nucleotide concentrations, in the co-sedimentation assays, were held in large excess over that of the protein, to ensure the desired nucleotide bound state of YqeH. We found that YqeH co-sediments with 30S stably in presence of GDPNP or GTP (Fig. 1E and F). On the contrary, in presence of GDP, the protein did not display a similarly strong interaction with 30S (Fig. 1G). Therefore, it appears that YqeH exhibits an apparent nucleotide (GTP/GDP) dependent 30S binding.

### Domains neighbouring the CPG-domain are important for ribosomal interactions

In an attempt to assess the role of N and C-terminal domains in ribosome binding, two deletion constructs – ΔN-YqeH lacking the N-terminal domain and ΔC-YqeH lacking the C-terminal domain, were created. Co-sedimentation experiments using these were conducted in the presence of GDPNP, as it promotes YqeH–30S interaction. In line with the RNA binding function assumed for the N-terminal domain [2], ΔN-YqeH failed to interact with 30S (Fig. 1I). This is consistent with the report, based on *in vivo* experiments, that disruption of Zn^2+^ co-ordination in the treble-clef motif produces a phenotype identical to that observed in the YqeH null mutant [10]. Interestingly, 30S interaction was abolished for ΔC-YqeH as well (Fig. 1H). In line with this, complementation experiments conducted in plants with ΔC-AtNOA1 too failed to rescue the wild type phenotype [16]. These experiments suggest an importance for both N and C-terminal domains in 30S interaction. To further assess their ability to bind ribosomes, we performed co-sedimentation experiments with stand-alone N-terminal Zn-finger domain and C-terminal PNR domain. However, we found that both of these display poor binding, if any (Fig. 1J and K). The fact that exogenous addition of missing domains, i.e., PNR domain to ΔC-YqeH and Zn-finger domain to ΔN-YqeH, did not restore 30S binding (data not shown) suggests the need for these domains to be covalently linked to the CPG-domain, which upon binding GTP evokes a strong 30S interaction. That both ΔN-YqeH and ΔC-YqeH display reduced GTP hydrolysis rates when compared to wild type (Table 1) further underscores the importance of these domains.

### YqeH binds RNA but does not display strand dissociation or annealing activity

**Fig. 2:**
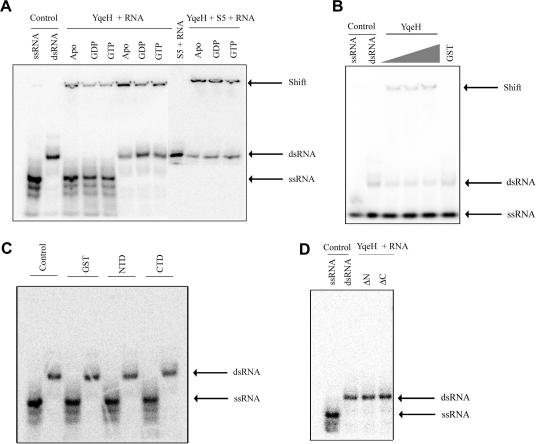
YqeH binds a non-specific RNA. EMSA was carried out with YqeH or the indicated domains, in presence of either ssRNA or dsRNA. The migration of ssRNA and dsRNA is shown as control in the lanes at the extreme left and is indicated by an arrow. A retarded migration of RNA in presence of YqeH is indicated by ’shift’. The nucleotide bound states of YqeH are indicated on the top. (A) Both ssRNA and dsRNA are retarded in the presence of YqeH. The dissociation of dsRNA into ssRNAs is not observed in nucleotide-free, GDP and GTP-bound forms. The presence of S5 is indicated above the lanes. The apparent reduction in the intensity of free dsRNAs in the presence of S5 when compared to the corresponding lanes containing YqeH alone suggests a potential increase in YqeH–RNA interactions (the last three lanes in the right). (B) Like in (A), the migration of ssRNA and the mixture of complementary ssRNAs is shown in the lanes at the extreme left as controls and is indicated by an arrow. Increasing concentration of YqeH (2.5, 5, 10 μM) is depicted by a triangle on the top. No annealing activity for YqeH was apparent. (C) EMSA carried out with GST, N (NTD) and C (CTD) terminal domains of YqeH (indicated above the lanes) showed no apparent shift in migration of ssRNA and dsRNA. (D) Also in the presence of deletion constructs (ΔN and ΔC-YqeH), no shift in dsRNA migration was observed.

YqeH null mutant displays an altered ribosome profile lacking a mature 30S subunit and severely compromised 16S rRNA stability [10,11]. On the other hand, deletion of YjeQ/YloQ, another cpGTPase that also binds 30S, did not show a similarly altered ribosome profile [4,5]. This indicated that YqeH may possibly participate in the early stages of 30S assembly by assisting 16S rRNA folding into the native state. To investigate such a possibility, the following experiments were designed. As proteins known to promote the folding of RNA bind non-specific RNA and exhibit strand dissociation or annealing activity (reviewed in [20]), two complementary single-stranded RNA with an arbitrary sequence and of length 21 bp were synthesized (for details see Supplementary material). YqeH interaction with single (ssRNA) and double-stranded RNA (dsRNA) was assayed using Electrophoretic Mobility Shift Assay (EMSA) to examine its ability to dissociate dsRNA (strand dissociation activity) and to promote association of complementary ssRNAs (annealing activity). In order to probe the effect of the bound nucleotides on RNA binding, nucleotide-free (apo), GDP and GTP-bound forms of YqeH were utilized. As shown in Fig. 2A, YqeH neither dissociates dsRNA into ssRNAs, nor promotes the annealing of complementary ssRNAs to dsRNA (Fig. 2B). On the other hand, YqeH binds both ssRNA and dsRNA in nucleotide-free, GDP and GTP-bound forms (Fig. 2A and B), suggesting RNA binding to be independent of the different nucleotide bound states of YqeH. To investigate if N-terminal Zn-finger domain and C-terminal PNR domains bind RNA; these experiments were repeated with the stand-alone domains. In line with the co-sedimentation assays (Fig. 1) conducted using these domains; here too, no apparent shift in mobility was evident (Fig. 2C). Similarly, the experiments with ΔN-YqeH and ΔC-YqeH, too, in presence of dsRNA, did not show any shift in RNA mobility (Fig. 2D).

Loh et al. [11] showed that GTP hydrolysis by YqeH is not stimulated in presence of 30S. Since YqeH binds ssRNA and dsRNA, GTP hydrolysis was assayed in their presence to probe whether RNA binding to YqeH influences GTP hydrolysis. It appears that ssRNA reduces GTP hydrolysis marginally, while dsRNA does not influence the activity (Table 1). Overall, these experiments negate an influence of RNA on GTP hydrolysis.

### S5 stimulates the GTP hydrolysis rates of YqeH

Gavin et al. [21] identified an association between LRC5, a yeast ortholog of YqeH localized in mitochondria and the mitochondrial small subunit ribosomal protein S5. Although we inquired a similar association between YqeH and S5 in *B. subtilis* using GST pull down and co-immuno precipitation experiments, these were not successful. However, interestingly, we observed an enhanced GTP hydrolysis in presence of S5 (Table 1) to anticipate a potential interaction between the two. We then examined if S5 influences ssRNA/dsRNA binding to YqeH. Although qualitative, EMSA experiments in presence of S5 reveal an apparent increase in YqeH–RNA interactions (Fig. 2A). Based on these, the implications of YqeH–S5 interaction are unclear and it would be interesting to explore this further in the context of 30S assembly.

## Discussion

The involvement of YqeH in the biogenesis of 30S ribosomal subunit is underscored by the absence of a matured 30S subunit in the YqeH deletion strain [10,11]. However, it was not known whether YqeH interacts with 30S. This study for the first time shows that YqeH exhibits a GTP/GDP dependent 30S binding, which may be achieved through the switch-II region in the G-domain, as proposed by us in an earlier work [2]. In YqeH, like the other cpGTPases, switch-II, a region important for GTP binding and hydrolysis, is relocated towards the C-terminus of the CPG-domain owing to the circular permutation [2]. As a consequence, the conformational changes associated with switch-II due to GTP/GDP binding could be propagated to the C-terminal domain and thus modulate ribosome binding [2]. Indeed, based on the recently determined structure of gsYqeH, the authors suggest that the GTP/GDP bound state modulates the relative three dimensional position of the C-terminal domain [19]. While 30S binding depends on GTP/GDP bound state, the observation that ΔN-YqeH and ΔC-YqeH do not bind 30S (Fig. 1), implicates that N and C-terminal domains, need to be covalently linked with the CPG-domain to offer a conformation suitable for ribosome binding. The role of the linker is underscored by the observation that exogenous supply of the missing N and C-terminal domains to ΔN-YqeH and ΔC-YqeH does not restore 30S binding (data not shown). Perhaps, the linkers connecting them sense the GTP/GDP state of CPG-domain, possibly via switch-II as discussed above, to ensure appropriate positioning of the C-terminal domain.

The 30S ribosome maturation involves the folding of 16S rRNA concomitant with the assembly of ribosomal proteins on rRNA [22,23]. Therefore, it appears that to direct the folding of rRNA into the native state under physiological conditions, accessory factors are required [23–25]. Some of these factors are expected to chaperone the folding of rRNA and possess a non-specific affinity towards RNA [20]. The altered ribosome profile coupled with 16S rRNA instability in YqeH deletion strains [10,11], probably indicates that 16S rRNA folding is incomplete. Since the assembly of ribosomal proteins onto 16S rRNA takes place in a hierarchical fashion, it is likely that any misfolding of the RNA in a particular step along the assembly pathway would prevent its association with a cognate ribosomal protein in the subsequent step. Such misfolded RNA may become a substrate for nucleases, thereby rendering it unstable. Here, the role of an RNA chaperone could be envisaged as one that would assist the folding of kinetically trapped misfolded RNA to its native state. However, with the current knowledge, how an RNA chaperone helps in folding is not well understood [20]. Given the altered ribosome profile and compromised stability of 16S rRNA in the YqeH deletion strain, is it possible that YqeH plays the role of an RNA chaperone during 30S assembly? *In vitro* studies using YqeH and non-specific ssRNA/dsRNA suggest neither strand dissociation nor annealing activities (Fig. 2A and B). While this negates an RNA chaperone activity, it raises the question how YqeH may influence 30S assembly.

Based on the commonality in the biochemical function of GTPases, Karbstein [25] proposes the following probable roles for GTPases in ribosome assembly (1) regulate the recruitment or displacement of ribosomal proteins onto the nascent ribosome; (2) act as a reversible placeholder to prevent premature ribosomal protein binding onto nascent ribosome; (3) sense the nutritional state of the cell in terms of GTP/GDP ratio; (4) promote conformational rearrangements within the nascent ribosome (RNA chaperone activity). While this study shows that YqeH lacks strand dissociation and annealing activity which allows one to exclude an RNA chaperone function (point 4), it would be interesting to explore the other possibilities.

## Figures and Tables

**Table 1:** Effect of RNA and S5 on GTP hydrolysis.

Construct	Specific activity (nM min^−1^ nM^−1^)
WT	0.2289 ± 0.0242
ΔN	0.0482 ± 0.024
ΔC	0.084 ± 0.0318
WT + ssRNA	0.1751 ± 0.0101
WT + dsRNA	0.2227 ± 0.0421
WT + S5	0.4699 ± 0.0301

Specific activity is represented as the amount of Pi released (nM) for a given concentration of enzyme (nM) for a certain time (min). Experiments were conducted in duplicates and were reproduced at least twice. The errors represent the standard deviation from the average. Values were corrected for the background intrinsic GTP hydrolysis.
