# Supplementary material for: Circularly permuted GTPase YqeH binds 30S ribosomal subunit: Implications for its role in ribosome assembly
Source: Biochem Biophys Res Commun. 2009 Sep 4;386(4):602–6. doi: 10.1016/j.bbrc.2009.06.078 (PMC2741578; doi:10.1016/j.bbrc.2009.06.078)
Supplement: Supplementary data [file mmc1.doc]

**Circularly permuted GTPase YqeH binds 30S ribosomal subunit:**

**Implications for its role in ribosome assembly**

Baskaran Anand, Parag Surana, Sagar Bhogaraju, Sushmita Pahari, Balaji Prakash*

Department of Biological Sciences and Bioengineering, Indian Institute of Technology, Kanpur 208016, India

* Author for correspondence

Email: [bprakash@iitk.ac.in](mailto:bprakash@iitk.ac.in)

**Supplementary Material**

**Cloning, Expression and Purification of YqeH and its derivatives**

The sequences encoding YqeH and YlqF from *B. subtilis* (see supplementary material: Table1) were PCR amplified using Pfu DNA polymerase (Fermentas) and cloned into a modified pGEX-4T1 vector (Amersham biosciences) using *NheI* & *XhoI* restriction sites. Proteins were overexpressed in *E.coli* BL21 (DE3). Cells were grown in LB broth and induced with 0.8mM IPTG (Sigma) when OD600reached 0.8. The cells were allowed to express the proteins for 3 hours, harvested and washed with 10mM sodium phosphate pH 7.4. The cell pellet was suspended in Lysis buffer (50mM Sodium Phosphate pH 7.4, 300mM NaCl, 10mM MgCl2­, 1mM DTT, 1mg/ml Lysozyme, Protease inhibitor cocktail (Sigma)) and lysed by repeated freeze-thaw cycles. Following incubation with 5μg/ml DNase and RNase for 1hr at 4°C, the cells were clarified by centrifugation at 40000g for 1hr and the supernatant was loaded onto a glutathione sepharose column (Amersham biosciences) which was pre-equilibrated with 5 column volumes (CV) of binding buffer (50mM Sodium Phosphate pH7.4, 300mM NaCl, 1mM DTT). The column was washed sequentially with 8 CV binding buffer, 5 CV buffer containing 50mM Tris-Cl pH 8, 650mM NaCl, 1mM DTT, and finally by 5 CV buffer containing 50mM Tris-Cl pH 8, 150mM NaCl, 1mM DTT. The protein was eluted with elution buffer (50mM Tris-Cl pH 8, 150mM NaCl, 1mM DTT, 30mM reduced Glutathione (Sigma)) and the concentration was estimated using BCA assay (Sigma). The protein was mixed with 50% glycerol, flash frozen in liquid nitrogen and stored at -80°C until required.

For the deletion constructs that lack the N (ΔN-YqeH; residues 64-366) and C-terminus (ΔC-YqeH; residues 1-224) respectively, as well as the stand-alone treble-clef Zn finger (residues 1-46) and PNR (residues 225-366) domains, the cloning, expression, and purification were carried out as above.

**Table 1**

**The list of primers used for cloning YqeH (or its derivatives), S5 and YlqF**

| **Constructs** | **Primers used*** |
| --- | --- |
| YqeH (WT) | FP: 5’- CTAGCTAGC ATGGAAAAGGTTGTTTG-3’  RP: 5’-CCGCTCGAG TCAAATTAAGGAACGCCG AAC-3’ |
| ΔN (residues 64-366) | FP: 5’-CTAGCTAGC GGTATTGGAGAAACGGAC TCTCTGG-3’  RP: 5’- CCGCTCGAG TCA AAT TAA GGA ACG CCG AAC-3’ |
| ΔC (residues 1-224) | FP: 5’- CTAGCTAGC ATGGAAAAGGTTGTTTG-3’  RP: 5’- CCGCTCGAG TTACCCTTGATGGTTGTT GATAATTCCCGGTG-3’ |
| ZnFinger domain (residues 1-46) | FP: 5’- CTAGCTAGC ATGGAAAAGGTTGTTTG-3’  RP: 5’- CCGCTCGAG TTAATTATAGTTTTTCAG TCTGAAGCAACGCTGG-3’ |
| PNR domain (residues 225-366) | FP: 5’- CTAGCTAGC ATGGCGCATTATGTC-3’  RP: 5’- CCGCTCGAG TCAAATTAAGGAACGCCG AAC-3’ |
| S5 (WT) | FP: 5’-GGAATTCCATATG ATGCGTCGTATTGACCCAAG-3’  RP: 5’- ATAAGAATGCGGCCGC TTATCCTAACA GTTCTTCTACAG-3’ |
| YlqF (WT) | FP: 5’ – CTAGCTAGC ATGACAATTCAATGGTTC CCGGGCC-3’  RP: 5’ – CCGCTCGAG TTACATCGTCGGCTGTTCAAATGAC-3’ |

* FP- Forward Primer; RP- Reverse Primer

The wild type represents the fulllength protein. The numbers inside the bracket give the domain boundaries for the deletion constructs

The recognition sites for *NheI* (GCTAGC), *XhoI* (CTCGAG), *NdeI* (CATATG) and *NotI* (GCGGCCGC) are underlined

**Co-Sedimentation assay**

*B. subtilis* 168 was grown at 30°C in LB broth till OD600 reaches 0.8. The cells were resuspended in buffer A (20mM Tris-Cl pH 8, 100mM NH4Cl, 10.5mM (CH3COO)2Mg, 0.5mM EDTA, 6mM 2-mercaptoethanol) and lysed in 5 freeze-thaw cycles. The cell debris were removed by centrifugation and the supernatant was layered on equal volume of buffer B (20mM Tris-Cl pH 8, 1M NH4Cl, 1.1M Sucrose, 10.5mM (CH3COO)2Mg, 0.5mM EDTA, 6mM 2-mercaptoethanol) and centrifuged for 5hrs at 250000g (4°C) using Sorvall TH660 rotor. The crude ribosome pellet was washed briefly with buffer A and resuspended in a small volume of buffer C (20mM Tris-Cl pH 8, 60mM NH4Cl, 10.5mM (CH3COO)2Mg, 6mM 2-mercaptoethanol), aliquoted and stored at -80°C following a snap freeze in liquid nitrogen.

The reaction mixture (100μl) consists of crude ribosomes (A260=2), 500nM protein (GST-YqeH or its derivatives; GST-YlqF; GST), 20mM Tris-Cl pH 8, 50mM NH4Cl, 1mM (CH3COO)2Mg, 1mM ZnCl2, 6mM 2-mercaptoethanol and 1mM nucleotide (GTP/GDP/GDPNP). Following incubation at 37°C for 30min, this mixture waslayered on a linear gradient of sucrose cushion (18-50%) that was manually made by a discontinuous loading of 50%, 32%, 27% and 18% sucrose in buffer D (20mM Tris-Cl pH 8, 60mM NH4Cl, 1.1mM (CH3COO)2Mg, 0.1mM EDTA, 6mM 2-mercaptoethanol) and centrifuged at 90000g for 10hrs at 4°C. Twenty fractions each containing 200μl were collected by gentle pipetting along the upper surface of the solution. Absorbancerecorded at 254nm was plotted for each fraction. The presence of 30S or 50S in these fractions was also assessed by the presence of 16S or 23S rRNA by extracting the RNA (using phenol-chloroform method) in each fraction and analyzing on a formaldehyde agarose gel (see supplementary material: Figure S1). Following this, the fractions were resolved on an SDS-PAGE and the presence of GST-YqeH (or GST-YlqF) was identified by using anti-GST antibody in a western blot. To probe the effect of GST on ribosome binding, co-sedimentation experiments were performed with GST and GST fused YlqF. Loss of ribosome binding to GST and occurrence of 50S binding to GST fused YlqF suggests that the ribosome binding is not hindered by GST (fig. 1c & 1d). Therefore, all co-sedimentation experiments were performed with the GST fusion constructs. Henceforth, YqeH refer to GST fusion constructs unless specified otherwise. These experiments were performed similarly for deletion constructs (ΔN-YqeH and ΔC-YqeH), treble-clef Zn finger and PNR domains.

**Figure S1**


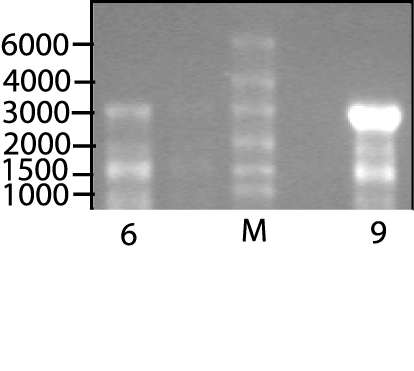


**Isolation of rRNAs from the ribosome fractions.**  The RNAs from the peak fractions 6 and 9 that correspond majorly to 30S and 50S, respectively, were isolated using phenol-chlorophorm extraction methods and resolved in denaturing agarose-formaldehyde gel. The RNA markers (M) and its corresponding sizes are indicated. The size of the 16S rRNA (30S) corresponds to around 1.5Kb and that of 23S rRNA (50S) it corresponds to around 3Kb.

**Purification of His tagged S5**

The sequences encoding S5 from *B. subtilis* were PCR amplified using Pfu DNA polymerase (Fermentas) and cloned into a pQE2 vector (Qiagen) using *NdeI* and *NotI* restriction sites. Proteins were overexpressed in *E.coli* BL21 (DE3). Cells were grown in LB broth at 37°C and induced with 0.2mM IPTG (Sigma) when OD600reached 0.8. The cells were allowed to express the proteins overnight at 18°C, harvested and washed with 10mM sodium phosphate pH 7.4. Cell lysis was done using ultrasonication with six cycles of alternating pulse (5 sec) and pause (30 sec). The supernatant of lysate was loaded onto a Ni2+ sepharose column (Amersham Biosciences) which was pre-equilibrated with 5 column volumes (CV) of binding buffer (50mM Sodium Phosphate pH7.4, 300mM NaCl, 6mM 2-mercaptoethanol). The column was washed with 8 CV binding buffer, 5 CV buffer containing 50mM Tris-Cl pH 8, 500mM NaCl, 6mM 2-mercaptoethanol, and finally by 5 CV buffer containing 50mM Tris-Cl pH 8, 150mM NaCl and 6mM 2-mercaptoethanol. The protein was eluted with a linear gradient of elution buffer (50mM Tris-Cl pH 8, 150mM NaCl, 1mM DTT, 300mM imidazole (Merck)) and the concentration was estimated using BCA assay (Sigma). The protein was aliquoted in small volume, flash frozen in liquid nitrogen and stored at -80°C until required.

**Nucleotide binding assays**

Nucleotide binding experiments were performed with nucleotide analogues carrying the N-methyl-3′-O-anthraniloyl (mant) fluorophore (Jena Biosciences). The experiments were carried out in 150μl reaction mixture in a buffer containing 20mM Tris-Cl pH 8, 50mM KCl, 1mM MgCl2 and 5mM 2-mercaptoethanol with 5μM YqeH (or GST) and 200nM mant-GDP or mant-GDPNP. After 10min incubation at 25°C, the fluorescent nucleotides were excited at 355nm and the emission (at 448nm) was monitored between 400 and 600nm using spectrofluorimeter (Perkin Elmer). The slit widths for both excitation and emission for GDPNP were kept at 10nm and for GDP they were kept at 5nm. In order to probe the effect of GST in GST fused YqeH, binding experiments were performed with GST. The absence of nucleotide binding to GST (fig. 1b) suggests that the fused GST does not hinder nucleotide binding to YqeH.

**GTPase assays**

The hydrolysis of GTP into inorganic phosphate (Pi) was measured in a calorimetric Malachite green assay as described [1]. The assay was performed in a buffer containing 20mM Tris-Cl pH 8, 50mM NH4Cl, 1mM (CH3COO)2 Mg, 6mM 2-mercaptoethanol with 400μM GTP, 375nM YqeH (or its derivatives). The reaction mixture (50 μL) was incubated at 37°C for 1hr after which the reaction was stopped by the addition of equal volume of malachite reagent. After 1 min, 34 μL of 30% sodium citrate was added to the mixture and the final volume was made up to 250 μL with distilled water. The reaction mixture was left at room temperature for 15min to allow color development, following which the absorbance at 630nm was measured using spectrophotometer (Perkin Elmer). The amount of inorganic phosphate released is estimated from the standard curve generated using known concentrations of potassium phosphate. In line with the nucleotide binding experiments, GST did not hydrolyse GTP (data not shown). GTPase assay to probe the influence of RNA contained 2 μl of 1mg/ml single or double stranded RNA and it contained 375nM S5 to probe the influence of S5. The assay was performed as described above.

**Electrophoretic mobility shift assay**

Two complementary oligonucleotides (21R(+) and 21R(-)) with an arbitrary sequence (see supplementary material: Table 2) of 21bp length were transcribed *in vitro* using T7 RNA polymerase (New England Biolabs), according to the manufacturer’s instruction. After removing the 5’ phosphates with calf intestinal alkaline phosphatase (Fermentas), the synthesized 21R(+) was 5’ end labeled using T4 polynucleotide kinase (New England Biolabs) and [γ−32P]ATP (BRIT, India). Labeled 21R(+) was then purified from nucleotides using ProbeQuant G-50 micro columns (Amersham Biosciences) and used directly for experiments with single stranded RNA. Double stranded RNA was prepared by slow cooling of equal amounts of labeled 21R(+) and unlabeled 21R(-) from 95°C to room temperature over the period of 3 hrs.

EMSA was performed with 5μM proteins (YqeH or its derivatives; GST; S5) in a 10μl reaction mixture consisting of 20mM Tris pH 8, 50mM KCl, 1mM MgCl2, 5mM 2-mercaptoethanol, 1U RNasin (Fermentas), 1mM of nucleotides (GTP/GDP) and 1μl of labeled (7000cpm/μl) single or double stranded RNA. After incubating at 37°C for 30min, the reaction was stopped by adding 10μl of stop buffer (20mM Tris pH 8, 50mM EDTA and 20% glycerol). The RNA was resolved in 12% native PAGE at 4°C by applying 200V for 1hr. The gel was dried and exposed to phosphor imager screen (Kodak) for visualization.

For annealing activity, the experiment was performed with complementary ssRNAs 21R(+) and 21R(-) that were not pre-annealed. Equal amounts of labeled 21R(+) and unlabeled 21R(-) were added to increasing concentrations of YqeH (2.5µM, 5µM, 10µM) and incubated for 2hrs at 37°C, following which the reaction was stopped by adding 10μl of stop buffer and resolved in 12% native PAGE at 4°C as mentioned above.

**Table 2**

**RNA template for *in vitro* transcription**

| **RNA Template** | **Sequence*** |
| --- | --- |
| T7(-)_21R(+) | 5’-ACTGCTAGAGATTTTCCACAT C CTA TAG TGA GTC GTA TTA-3’ |
| T7(-)_21R(-) | 5’-ATGTGGAAAATCTCTAGCAGT C CTA TAG TGA GTC GTA TTA-3’ |

21R(+) - 5’-ATGTGGAAAATCTCTAGCAGT-3’ (labeled with P32 at 5’)

21R(-) - 5’-ACTGCTAGAGATTTTCCACAT- 3’ (unlabeled)

* The complementary sequence of T7 promoter is underlined

**References**

[1] A.A. Baykov, O.A. Evtushenko, and S.M. Avaeva A malachite green procedure for orthophosphate determination and its use in alkaline phosphatase-based enzyme immunoassay. Anal Biochem 171 (1988) 266-270.
